# Reduction of nicotine content in tobacco through microbial degradation: research progress and potential applications

**DOI:** 10.1186/s13068-024-02593-3

**Published:** 2024-12-18

**Authors:** Zi-Jia Li, Dong-Dong Yang, Zhi-Yun Wei, Jie Huang, Yi-Qian Chi, You-Xuan Lu, Feng-Wei Yin

**Affiliations:** 1 School of Life Sciences, Taizhou University, Taizhou, 318000 Zhejiang People’s Republic of China; 2 School of Food Science and Pharmaceutical Engineering, Nanjing Normal University, Nanjing, 210000 People’s Republic of China; 3 Taizhou Key Laboratory of Biomass Functional Materials Development and Application, Taizhou University, Taizhou, 318000 Zhejiang People’s Republic of China

**Keywords:** Tobacco, Nicotine-degrading microorganisms (NDMs), Biotechnology, Novel tobacco products

## Abstract

Originally native to South America, tobacco and is now distributed worldwide as a major cash crop. Nicotine is the main harmful component of tobacco leaves, cigarette smoke and tobacco waste, which severely affects not only the flavor of the tobacco leaf, but also causes great damage to human health. As the anti-smoking movement continued to grow since the 1950s, and consumers become more aware of their health and environmental protection, the world tobacco industry has been committed to research, develop and produce low nicotine cigarette products with relatively low risk to human health. Among various approaches, the use of microorganisms to reduce nicotine content and improve tobacco quality has become one of the most promising methods. Due to increasing interest in nicotine-degrading microorganisms (NDMs), this article reviews recent reports on NDMs, nicotine-degrading enzymes, regulation of nicotine-degrading bacterial consortia and optimization of fermentation conditions, aiming to provide updated references for the in-depth research and application of microorganisms for the degradation of nicotine.

**Graphical Abstract:**

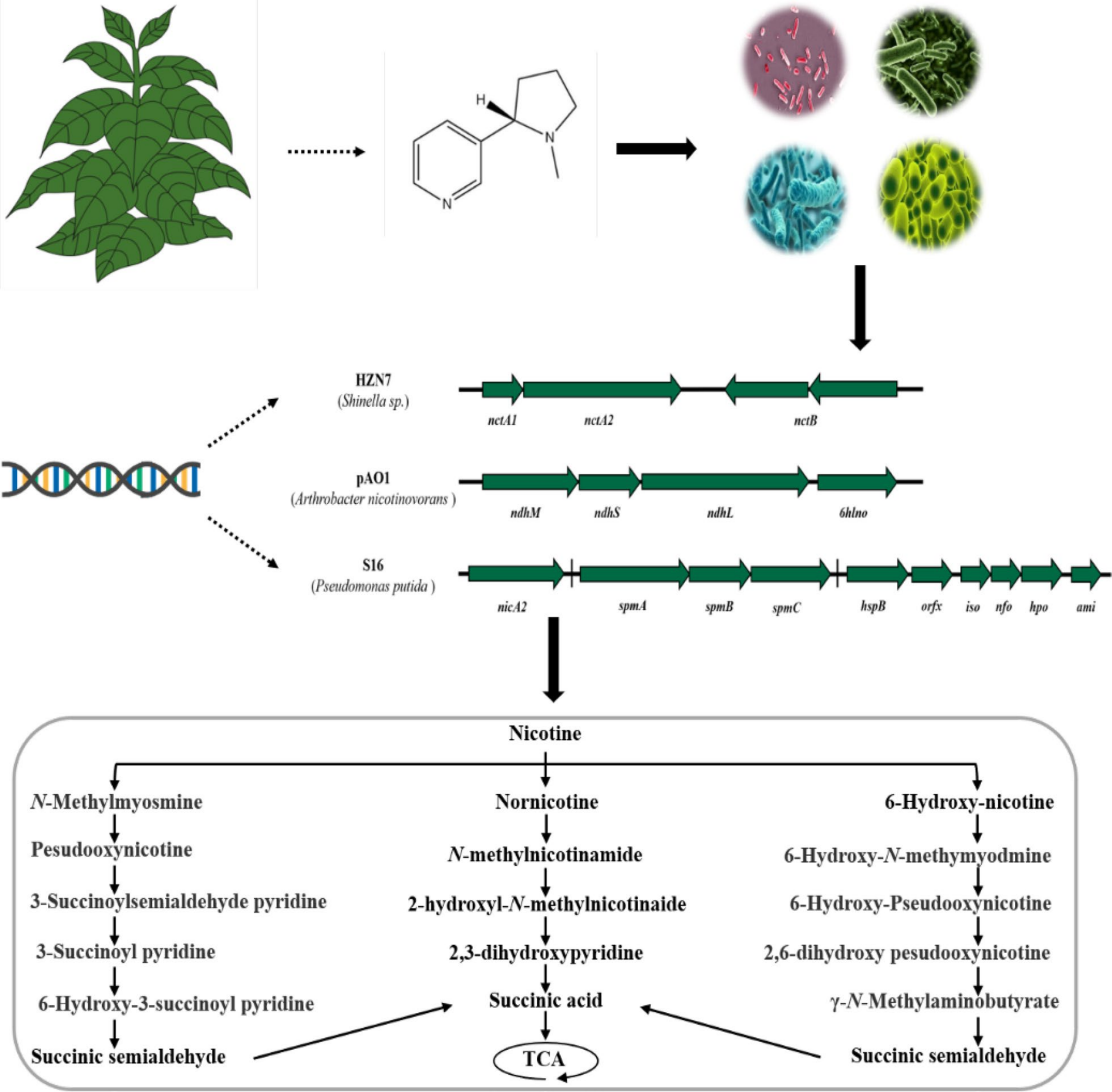

## Background

Since its earliest domestication in South America, tobacco has been cultivated in over 125 countries, generating billions of dollars in revenue each year and playing a vital role in the economy of many countries [1]. However, due to a lack of advanced processing technologies, the content of harmful substances in tobacco is high, which has caused serious damage to human health and has led to campaigns against tobacco use [2, 3]. The characteristic harmful substances of tobacco mainly include nicotine, N-nitrosamines (TS-NAs) and tar. Nicotine (3-(1-methyl-2-pyrrolidinyl)pyridine), a heterocyclic compound with a pyridine and a pyrrolidine ring moiety, is the major harmful component in tobacco leaves, cigarette smoke and tobacco waste [4]. Nicotine is a central nervous stimulant that can cause physiological and psychological dependence, which has led to its classification as an addictive substance. Moreover, it can also lead to tobacco-related lung cancer and peripheral artery disease, causing irreversible damage to the human body. A large amount of high nicotine tobacco is produced every year, which not only seriously affects the flavor of tobacco products, but also increases their harmfulness [5–12]. Therefore, reducing the content of nicotine in tobacco is of great importance for maintaining human health and protecting the ecological environment. With the constant advancement of science and technology, microbiology has been widely used in the fields of food, medicine and health care products, and also provides a premise to improve the quality of tobacco [13–18]. Microbial treatment is an effective way to reduce the content of nicotine in tobacco and the environment, while preventing the loss of tobacco flavor, and also improving the quality of smoke, increasing the efficiency of tobacco resources, and more importantly, reducing the damage of nicotine to human health, showing great potential for development. To date, a large amount of basic and applied research work has been carried out in the use of microorganisms for nicotine degradation [15, 19–29]. In particular, some progress has been made in recent years in the study of molecular mechanisms such as key enzymes and related genes for nicotine degradation. This review summarizes recent reports on nicotine-degrading microorganisms, nicotine-degrading enzymes, regulation of nicotine-degrading bacterial consortia, and optimization of fermentation conditions, with the aim of promoting basic microbiological research in the tobacco industry.

## Nicotine-degrading microorganisms

To date, researchers have developed physical, chemical and biological methods for nicotine degradation. However, physical and chemical methods have the disadvantages of low degradation efficiency and complex equipment. By contrast, microbial methods use a variety of nicotine-degrading microorganisms and enzymes, which are cheaper, more efficient, and more ecologically friendly [21, 24, 30]. Some large tobacco companies, such as B&W Tobacco and British American Tobacco, have been using microorganisms to degrade nicotine in tobacco for a long time. Microorganisms that degrade nicotine have been mainly isolated and screened from the surface of tobacco leaves or soil [31]. They can usually utilize nicotine as the sole carbon, nitrogen and energy source for their growth [32, 33].

**Fig. 1:**
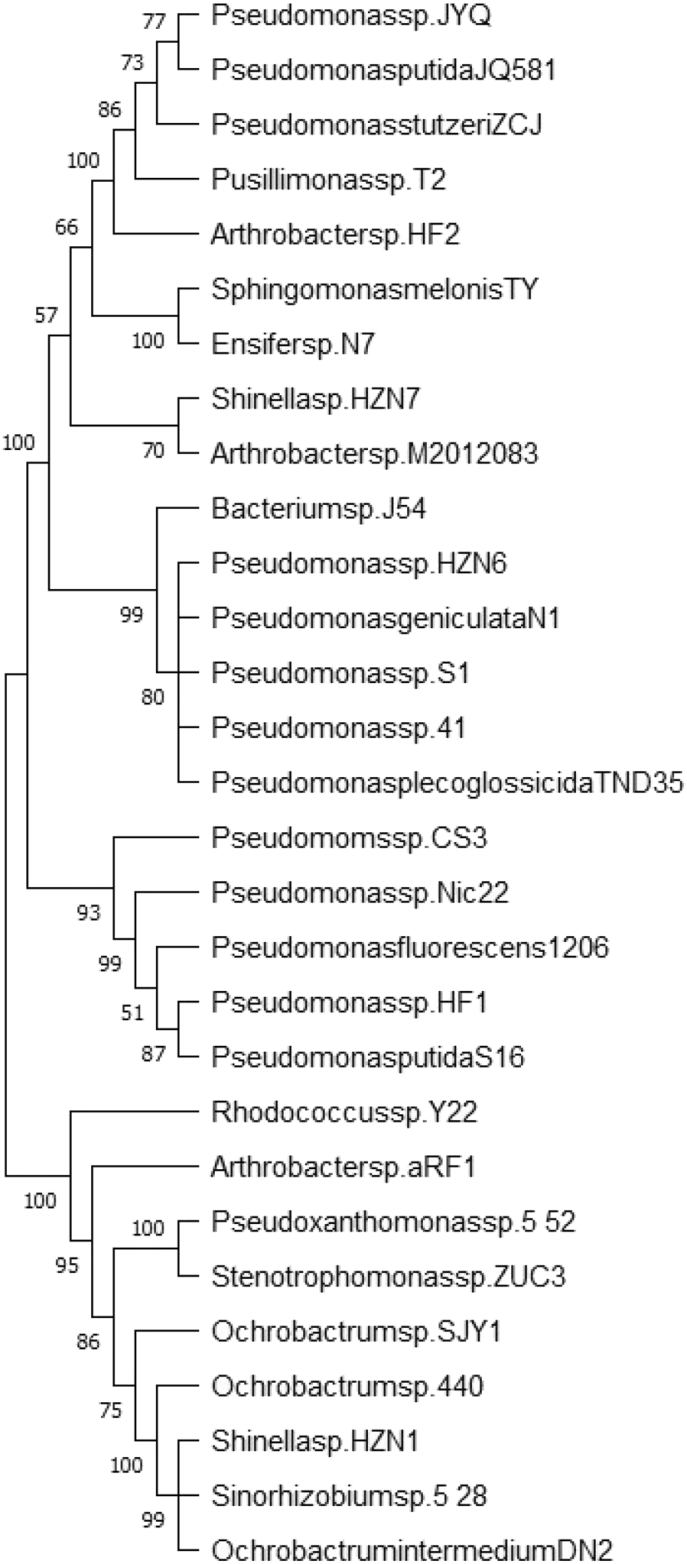
Phylogenetic relationships of nicotine-degrading bacteria and related species were analyzed using 16S rRNA gene sequences. A neighbor-joining tree was constructed with MEGA 5.0

Since Reid et al. first discovered in 1944 that nicotine-degrading bacteria and molds are present on the surface of cigar tobacco leaves, a large number of nicotine-degrading microorganisms were screened, isolated and identified [34]. For example, Gutierrez et al. isolated the *Enterobacter cloacae* strain E-15040 from the surface of dried tobacco leaves, which was able to degrade nicotine [35]. In 2004, *Pseudomonas putida* S16 was isolated from soil ( Shandong, P.R. China), and exhibited a high nicotine degradation capacity, degrading 4 g L^−1^ nicotine in 13 h and tolerating concentrations of up to 6 g L^−1^ nicotine [36]. Similarly, Yuan et al. [37] used enrichment culture technique to isolate the highly efficient nicotine degrading strain *Ochrobactrum intermedium* DN2 (Fujian, P.R. China), which had high degradation activity in the temperature range of 30 ~ 40 °C and pH 6.0 ~ 9.0, reaching a degradation efficiency of 97.56% after 36 h. In 2008, *Pseudomonas* sp. Nic22 was isolated and its crude enzyme extract was found to not only reduce the nicotine content, but also improve the quality of tobacco leaves (Zimbabwe and, P.R. China) [38]. In addition, the *Agrobacterium* sp. strain S33, which could degrade 3 g L^−1^ nicotine in 1.5 h, was isolated from soil in a tobacco-growing area by Wang et al. (Shandong, P.R. China) [39]. In 2012, *Ochrobactrum* sp*.* 4–40, isolated from tobacco leaves, was discovered to be effective in degrading nicotine in tobacco (Mile, Yunnan, P.R. China) [40]. A few years later, *Pseudomonas aeruginosa* TND35 was revealed to degrade 4.92 g L^−1^ of nicotine in 44 h by Raman et al., and the degradation rate of nicotine was increased during accelerated growth of bacterial cells (Ottanchathiram, Tamil Nadu, India) [41]. In recent years, many more novel nicotine-degrading strains have been isolated. Particularly surprisingly, Chen et al. recently discovered for the first time that the gut bacterium *Bacteroides xylanisolvens* can effectively degrade nicotine in the human gut and identified the novel nicotine-metabolizing enzyme NicX and its metabolites [42]. As shown in Table 1 and Fig. 1. In general, the bacteria that can degrade nicotine mainly belong to the genera *Pseudomonas* [32], *Arthrobacter* [32], and *Ochrobactrum intermedium* [37]. The main nicotine-degrading fungi are *Cunninghamella echinulata* [41], *Microsporum gypseum*, *Streptomyces griseus*, *S. platensis* and *Pellicularia filamentosa* [43]. These microorganisms play an important role in tobacco processing and tobacco waste treatment. For example, *Pseudomonas* spp. are highly favored by researchers as they produce many intermediate products with high added value in the process of nicotine degradation. To date, a number of wild-type strains capable of degrading nicotine have been widely used to degrade nicotine and treat waste from tobacco manufacturing processes [44].

**Table 1:** List of reported nicotine degrading strains

Classification	NDMs	Sources	Treatment conditions	Nicotine-degrading efficiency	References
Commonly used strains	*Enterobacter cloacae* E-150	Tobacco leaves	–	–	[35]
	*Pseudomonas putida* S16	Tobacco rhizosphere soil	30 °C, pH 7.0, 13 h	4 g/L,	[36]
	*Ochrobactrum intermedium* DN2	Tobacco rhizosphere soil	30–37 °C, pH 7.0, 36 h	0.5 g/L,	[37]
	*Pseudomonas* sp. Nic22	Tobacco rhizosphere soil	30–34 °C, pH 6.5, 48 h	2.87 g/L	[38]
	*Agrobacterium* sp. strain S33	Tobacco rhizosphere soil	30 °C, pH 7.0, 3 h	1.5 g/L	[39]
	*Ochrobactrum* sp. 4–40	Tobacco rhizosphere soil	–	–	[40]
	*Pseudomonas plecoglossicida* TND35	Tobacco rhizosphere soil	30 °C, pH 7.0, 12 h	3.0 g/L	[41]
Other bacterial species	*Acinetobacter* sp. DN12	Tobacco rhizosphere soil	28 °C, pH 6.0, 14 h	1.0 g/L	[96]
	*Acinetobacter* sp. TW	Tobacco waste	30 °C, pH 7.0, 12 h	1.0 g/L	[97]
	*A. nicotianae* K9	Tobacco rhizosphere soil	30 °C, 64 h	1.0 g/l	[66]
	*Arthrobacter* sp. HF-2	Tobacco waste	30 °C, pH 7.0, 43 h	0.7 g/L	[98]
	*Arthrobacter* sp. M2012083	Tobacco waste	–	–	[99]
	*Arthrobacter* sp. aRF-1	Tobacco rhizosphere soil	30 °C, pH 7.0, 48 h	5.0 g/L	[100]
	*Arthrobacter* sp. AH14	Tobacco rhizosphere soil	34 °C, pH 6.5, 120 h	6.0 g/L	[101]
	*Bacillus* sp. J54	Tobacco leaves	–	–	[102]
	*Ensifer* sp. N7	Tobacco rhizosphere soil	28 °C, pH 7.0, 48 h	3.35 g/L	[103]
	*Ochrobactrum* sp. SJY1	Wastewater	30 °C, pH 7.0, 10 h	4.0 g/L	[104]
	*Paenarthrobacter nicotinovorans* pAO1	–	125 h	3.4 g/L	[105]
	*Rhodococcus* sp. Y22	Tobacco rhizosphere soil	28 °C, pH 7.0, 52 h	1.0 g/L	[106]
	*Pseudomonas* sp. HF-1	Tobacco waste	30 °C, pH 6.5–7.5, 25 h	1.3 g/L	[63]
	*Pseudomonas* sp. ZUTSKD	TWE	30 °C, pH 7.0, 12 h	1.55 g/L	[107]
	*Pseudomonas* sp. HZN6	Sludge	30 °C, pH 7.0, 8 h	0.5 g/L	[108]
	*Pseudomonas* sp. CS3	Tobacco rhizosphere soil	30 °C, pH 7.0, 24 h	1.0 g/L	[75]
	*Pseudomonas* sp. JY-Q	TWE	30 °C, pH 7.0, 24 h	5.0 g/L	[109]
	*Pseudomonas* sp. S-1	Tobacco waste	30 °C, pH 7.0, 12 h	0.4 g/L	[110]
	*Pseudomonas* sp. 41	Tobacco rhizosphere soil	30 °C, pH 6.4, 24 h	1.3 g/L	[56]
	*Pseudoxanthomonas* sp. 5–52	Tobacco leaves	28 °C, pH 7.0, 28 h	1.0 g/L	[40]
	*Pseudomonas putida* JQ581	Marine sediments	2 h	0.6 g/L	[111]
	*Pseudomonas geniculata* N1	Tobacco waste	30 °C, pH 6.5, 6.5d	1.0 g/L	[112]
	*Pseudomonas fluorescens* 1206	Tobacco rhizosphere soil	24 h	1.0 g/L	[113]
	*Pseudomonas stutzeri* ZCJ	Tobacco leaves	37 °C, pH 7.4, 24 h	1.3 g/L	[79]
	*Pusillimonas* sp. T2	Activated sludge	30 °C, pH 7.0, 8 h	0.5 g/L	[114]
	*P. putida* J5	Tobacco leaves	30–34 °C, pH 6.5, 52 h	2.89 g/L	[115]
	*P. stutzeri*	Tobacco leaves	37 °C, pH 7.5, 36 h	2.5 g/L	[79]
	*Sphingomonas melonis* TY	Tobacco waste	30 °C, pH 7.0, 18 h	1.0 g/L	[97]
	*Shinella* sp. HZN1	Activated sludge	30 °C, pH 7.0, 9 h	0.5 g/L	[116]
	*Shinella* sp. HZN7	Sludge	30–35 °C, pH 6.5–8.0, 24 h	0.23 g/L	[116]
	*Sinorhizobium* sp. 5–28	Tobacco rhizosphere soil	28 °C, pH 7.0, 28 h	1.5 g/L	[40]
Fungi	*Aspergillus oryzae* 112,822	Tobacco leaf	28 °C, pH 6.5, 40 h	2.19 g/L	[51]
	*Cunninghamella echinulata* IFO-4444	–	13d	0.54 g/L	[117]
	*Pellicularia filamentosa* JTS-208	Tobacco plant	20d	0.04 g/L	[117]
	*Microsporum gypseum*	–	–	–	[16]

## Catabolism of nicotine in microorganism

With the continuous development of science and technology, as well as the improvement of the level of instrumental analysis, in recent years, studies went beyond the screening, isolation and identification of nicotine-degrading microorganisms, and have further explored the genetic background of their special metabolic processes. As a result, the specific reaction steps of nicotine degradation and the enzymes involved in catalysis have gradually been clarified in some strains [14, 28, 45–50].

Omics technologies have greatly accelerated the comprehensive understanding of nicotine metabolism and regulation in microorganisms. Based on this, three biodegradation pathways of nicotine have been summarized [43, 51]. One is the pyrrolidine pathway present in *Pseudomonas* spp. and *Cunninghamella echinulata*. The second is the pyridine pathway found in *Arthrobacter sp.* The third is the Me pathway present in tobacco plants and fungi. Among these three metabolic pathways, the pyridine pathway based on plasmid pAO1-mediated degradation of nicotine by *Arthrobacter nicotinovorans* is the most well-studied.

**Fig. 2:**
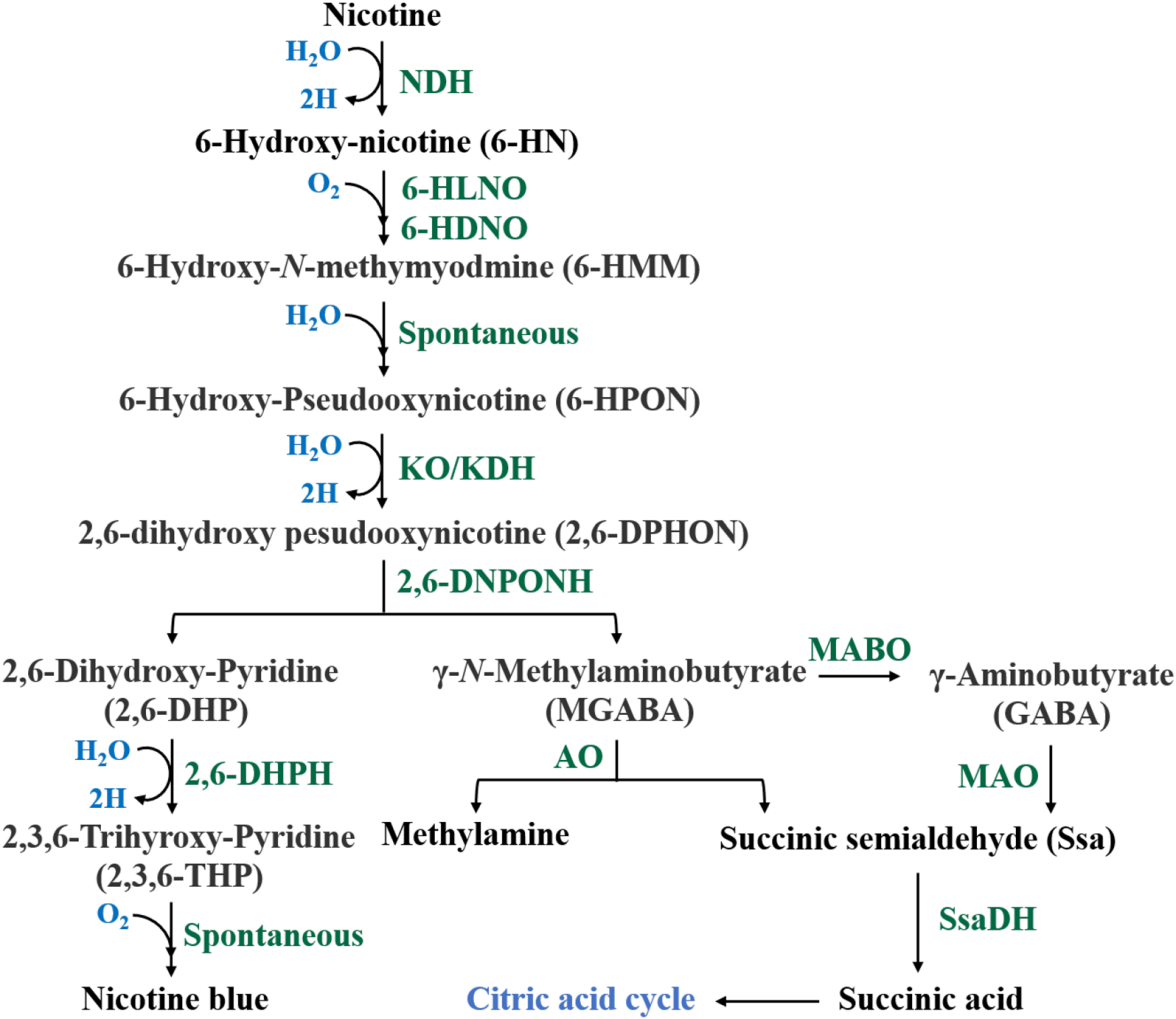
Pyridine pathway of nicotine degradation in *Arthrobacter* sp. involves key enzymes: nicotine dehydrogenase (NDH), 6-hydroxy-l-nicotine oxidase (6-HLNO), 6-hydroxy-d-nicotine oxidase (6HDNO), ketone oxidase (KO), ketone dehydrogenase (KDH), 2,6-dihydroxy-pseudooxynicotine hydrolase (2,6-DHPONH), 2,6-dihydroxypyridine-3-hydroxylase (2,6-DHPH), γ-N-methylaminobutyrate oxidase (MABO), monoamine oxidase (MAO), amine oxidase (AO), and succinic semialdehyde dehydrogenase (SsaDH)

The pyridine pathway was first studied in detail by Baitsch et al. [14] and Brandsch et al. [13], who found that the biodegradation of nicotine is accomplished stepwise by several different enzymes [13]. A more detailed summary of the pathway is as follows (Fig. 2): (1) nicotine enters bacterial cells and is converted into 6-hydroxynicotine (6-HN) by nicotine dehydrogenase (NDH). Then, 6-HN can be processed to synthesize pyridine, which is an important precursor compound for industrial synthesis of painkillers [15, 27, 52]. (2) 6-Hydroxynicotine oxidase (6-HNO) catalyzes the dehydrogenation and oxidation of 6NH to 6-hydroxy-N-methylmyosmine (6-HMM), which is unstable and is spontaneously hydrolyzed to produce 6-hydroxy-pseudooxynicotine (6-HPON) [29]. (3) The pyridine ring is further opened to produce 2,6-dihydroxy pseudooxynicotine (2,6-DPHON) [53]. (4) The side chain of 2,6-DHPON is broken and cleaved by 2,6-dihydroxy pseudooxynicotine hydrolase (DHPONhl) to produce 2,6-dihydroxypyridine (2,6-DHP) and γ-N-methyl-aminobutyrate (MGABA). (5) Under the catalysis of 2,6-dihydroxypyridine-3-hydroxylase (DHPhl), 2,6-DHP is transformed into 2,3,6-trihydroxypyridine (2,3,6-THP). Finally, in the presence of oxygen, 2,3,6-THP is spontaneously oxidized and dimerizes to form nicotine blue and several other organic acids [54].

Ganas et al. [47] conducted a deeper molecular study of *Arthrobacter nicotinovorans*, and identified the positions of some genes encoding key enzymes related to nicotine degradation in the Nic gene cluster, achieving a significant breakthrough in the understanding of microbial nicotine degradation at the molecular level. Further studies revealed that nicotine can activate nicotinic acetylcholine receptors (nAChRs) in the ventral tegmental area (VTA) of the midbrain, where dopaminergic (DAergic) neurons are enriched, and that nicotine-induced activation of corticotropin reward circuits in the midbrain may trigger addiction [55]. Accordingly, these receptors may provide new molecular targets for smoking cessation treatment.

**Fig. 3:**
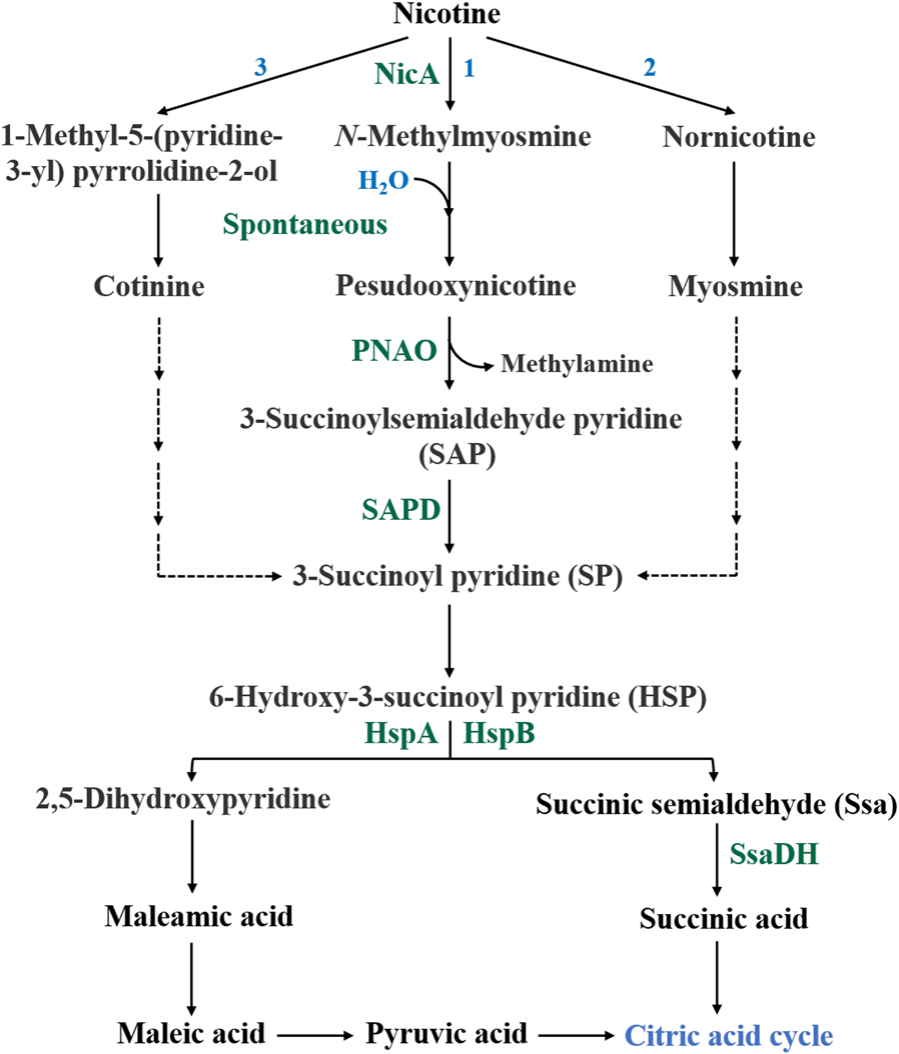
Pyrrolidine pathways of nicotine degradation have been identified in various *Pseudomonas* species: *Pseudomonas* sp. HZN6, *Pseudomonas* sp. HF-1, *Pseudomonas* sp. Nic22, and *Pseudomonas* sp. CS3. Key enzymes involved include nicotine oxidoreductase (NicA), pseudooxynicotine amine oxidase (PNAO), 3-succinoylsemialdehyde pyridine dehydrogenase (SAPD), and 6-hydroxy-3-succinoyl pyridine hydroxylases (HspA and HspB)

Nicotine-degrading *Pseudomonas* spp. were isolated from soil as early as 1954 and were extensively studied (Fig. 3) [56]. Recently, many researchers [36, 46, 57, 58] conducted numerous excellent studies in elaborating the metabolic pathways and related mechanisms of nicotine degradation by *Pseudomonas putida* S16. On the basis of these studies, Tang et al. cloned, sequenced, and expressed a 4879 bp-long nicotine metabolizing gene cluster from the genome of *Pseudomonas putida* S16 by cell fusion. The intermediate metabolites produced by the recombinant engineered cells were the same as those produced by the wild-type strain, and were derived from the pyrrole pathway [48]. In 2015, the nicotine-degrading enzyme NicA2, a flavoprotein derived from *Pseudomonas putida*, was found to have special biochemical properties and degrade nicotine into non-addictive substances, making Nic A2 a promising candidate in the field of nicotine addiction treatment [59]. In 2019, Thisted et al. [60] found that mutating the relevant active site of the *P. putida* S16-derived Nic A2 enzyme could significantly increase its catalytic activity, providing an approach to optimize nicotine degradation by increasing enzyme activity. However, the nicotine degradation efficiency is not high due to the fact that nicotine-degrading microorganisms tend to preferentially use glucose in the fermentation broth as a carbon source when degrading nicotine in tobacco waste extracts. To address this problem, Zhang et al. [61] used homologous recombination to knock out all five genes related to glucose metabolism. The resulting *Pseudomonas* sp. strain JY-Q/5∆ could completely degrade 0.8 g L^−1^ of nicotine in 5% tobacco waste extract dilution within 24 h, expanded applications of *Pseudomonas* spp. in practice.

Notably, tobacco plants and fungi also play distinct roles in nicotine degradation, with mechanisms that differ significantly from those of bacteria. For example, fungi such as *Aspergillus oryzae* and *Phanerochaete chrysosporium* primarily degrade nicotine through the demethylation pathway (Me pathway). In this process, nicotine is gradually converted into intermediates such as N-methylmyosmine and pseudooxynicotine, eventually producing less toxic compounds like nicotinic acid. These fungi also secrete various enzymes, such as lignin peroxidase, to further break down other components of tobacco, including lignin. Compared to bacterial pathways that rely on oxidoreductases and hydrolases, the fungal degradation pathways demonstrate unique evolutionary adaptations in environmental versatility and degradation diversity, giving fungi an advantage in processing complex biomass and generating diverse chemical products. In addition, beyond bacteria and fungi, other microbial populations have also shown high efficiency in nicotine degradation. For instance, *Ochrobactrum intermedium* DN2 degrades nicotine primarily through a variant pyridine and pyrrolidine pathway (VPP pathway), which integrates upstream and downstream reactions of the classic pyridine and pyrrolidine pathways, enabling the strain to utilize nicotine as the sole source of carbon, nitrogen, and energy, ultimately converting it into non-toxic or low-toxicity products [62].

**Fig. 4:**
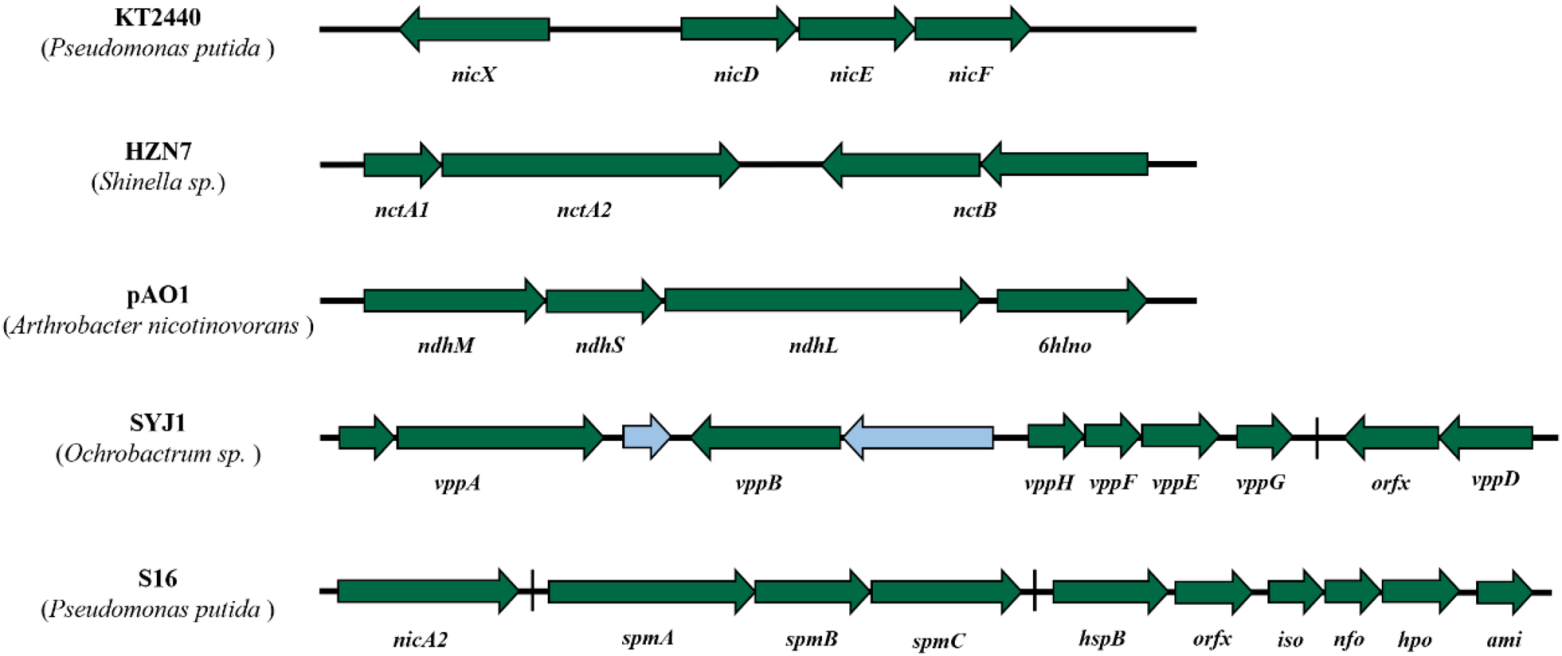
Some of the reported nicotine degradation gene clusters. All gene clusters contain genes encoding key enzymes. The presence and varied arrangement of auxiliary genes indicate evolutionary divergence and functional specialization among the nicotine-degrading bacteria

In summary, it can be seen that the microbial degradation of nicotine is a complex metabolic process, and there are still some metabolic steps and enzymes have not been elucidated. Future studies should aim to identify new nicotine degradation enzymes from different sources and co-express them in nicotine-degrading strains (Fig. 4), or to knock out the genes that inhibit nicotine degradation, so that we can quickly and effectively obtain genetically engineered strains with stronger degradation ability than wild-type strains, which can maximize their potential to improve tobacco quality and protect the environment.

## Regulation of nicotine degradation by microbial consortia

The fermentation of tobacco is a sophisticated process that involves spontaneous oxidation, as well as microbial and enzymatic action. However, reducing the nicotine content to a reasonable level is an important factor irrespective of the mechanism. In addition to reducing the nicotine content, some microorganisms can also act on the sugars and proteins in the tobacco leaves, converting them into alcohols, esters, various organic acids and amino acids, which neither destroy the original good quality of the tobacco leaves, nor increase the aroma of the tobacco leaves. Therefore, using microorganisms to solve the problem of excessive nicotine content in tobacco leaves is an efficient, environmentally friendly and low-cost approach.

Related research and applications have been carried out by tobacco researchers from various countries since the 1950s. A pioneering study published by Frankenburg et al. showed that the natural fermentation process of cigar tobacco was accompanied by a considerable degradation of nicotine [63]. In 1957, he isolated microorganisms from the surface of three types of tobacco seeds for nicotine degradation research. After degradation, oxamic acid, formamide, trace amounts of malonic acid and succinic acid can be generated [64]. Geiss et al. [21] placed several bacteria (such as *Cellulomonas* sp. and *P. putida*) in media containing nicotine and nitrate and used these cultures to treat white-ribbed tobacco under aerobic or anaerobic conditions. The levels of nitrate and nicotine in the treated tobacco were found to be reduced, and when used to treat tobacco products, the amount of nitrogen oxides, hydrogen cyanide, and nicotine in the smoke was diminished. At the same time, there was no loss of desirable flavor or smoking properties. Subsequently, Meher et al. [24] reduced chemical oxygen demand (COD), nicotine, and biological oxygen demand (BOD) in tobacco by 60%, 75%, and 80%, respectively, using fermentation with a mixture of methanogenic bacteria. Moreover, the microflora can also play an important role in reducing nicotine content when dealing with tobacco waste. For example, compost fermentation of tobacco solids was investigated by Briski [65] and it was revealed that at the end of composting, the nicotine in tobacco waste was reduced by 80%. The dominant microorganisms in the fermentation process were fungi and thermophilic bacteria, mainly yeasts, *Pseudomonas*, *Bacillus* and thermophilic actinomycetes.

Currently, *Pseudomonas* spp. are the most widely used bacteria in the degradation of nicotine to improve the quality of tobacco leaves [32, 38, 66]. Because *Pseudomonas* spp. has the highest degradation capacity[67]. In addition, optimizing culture conditions and utilising metabolic engineering strategies can significantly enhance the efficiency of nicotine degradation in tobacco leaves and products. Bacteria play a critical role in this process, particularly species such as *Bacillus subtilis*, *Bacillus coagulans*, *Bacillus circulans*, *Bacillus megaterium*, and *Bacillus thuringiensis*, which are commonly used to treat tobacco leaves and products. These bacteria help to generate desirable aromas in tobacco, thereby improving the sensory qualities and smoking characteristics of the tobacco [68–71]. In addition to these bacteria, fungi, yeasts, and thermophilic actinomycetes are also crucial in the tobacco degradation process. Fungi like *Aspergillus oryzae* and *Phanerochaete chrysosporium*, as well as certain yeasts, secrete a variety of enzymes, such as lignin peroxidase and cellulase, which break down complex organic compounds in tobacco, including lignin, cellulose, and hemicellulose. The secretion of these enzymes not only aids in nicotine degradation but also enhances the generation of tobacco aromas and produces a range of potentially valuable by-products during the degradation process. In contrast, thermophilic actinomycetes, such as members of the genera *Thermomonospora* and *Streptomyces*, are highly effective at degrading nicotine and other harmful substances in tobacco under high-temperature conditions. This makes them particularly valuable in the treatment of high-temperature composting and other thermally processed tobacco waste [13, 47, 51, 62, 71].

Thus, in the regulation of microbial consortia for tobacco degradation, bacteria, fungi, yeasts, and thermophilic actinomycetes each play unique and complementary roles. By combining the strengths of these different microorganisms, the overall efficiency of tobacco leaf and product degradation can be significantly improved, while also enhancing the quality of tobacco products to better meet market demands. Future research can explore several feasible strategies to further enhance the efficiency of tobacco degradation. First, optimizing microbial synergy is crucial; studying the optimal combinations of different microbial communities, such as co-cultivating fungi and bacteria, can lead to synergistic effects, maximizing degradation efficiency by complementing each other's capabilities in breaking down various organic components. Second, genetic engineering of key microorganisms could enable the expression of more efficient enzymes or metabolic pathways involved in nicotine degradation. By employing gene editing techniques, it may be possible to enhance the ability of microorganisms to process recalcitrant components in tobacco. In addition, precise control of environmental conditions during fermentation, including temperature, pH, and oxygen concentration, can promote the selective growth of advantageous microbial communities, thereby improving overall degradation efficiency. For instance, dynamically adjusting fermentation temperatures can fully exploit the characteristics of thermophilic actinomycetes and other microorganisms. Finally, there is potential to further develop the valuable by-products generated during microbial degradation into commercially viable products, such as flavor compounds, chemical precursors, or bioenergy, thereby enhancing the economic viability of the entire degradation process.

## Optimization of fermentation conditions

During tobacco fermentation, there are regular changes in the microbial community, and the number of microorganisms is significantly correlated with enzyme activity, indicating that the quality of tobacco may be related to the enzymes secreted by microorganisms and their activities.

Temperature and pH are the two most important factors affecting microbial growth and enzymatic activities [72–74]. In general, a higher microbial biomass will result in a lower residual nicotine content. However, the optimal culture conditions for nicotine degradation vary among different nicotine-degrading microorganisms. For example, the optimal nicotine degradation temperature of *Pseudomonas* sp. CS3 was 30 °C and the nicotine degradation ability was sharply decreased at temperatures above 37 °C or below 25 °C. Similarly, the nicotine-degradation activity of *Pseudomonas* sp. Nic22 was stable at 30–34 °C, but greatly decreased at temperatures below 28 °C or above 34 °C. However, he optimal temperature for nicotine degradation by *Pseudomonas stutzeri* ZCJ was 37 °C. By contrast, *Shinella* sp. HZN7 degraded nicotine efficiently within 30–35 °C. Finally, *P. putida* S16 had a high nicotine degradation efficiency at 30 °C [75]. In addition to temperature, the pH also affects the growth of microorganisms, which will in turn affect the degradation of exogenous compounds. Wang et al. [76] reported that the most favorable pH for degradation of nicotine by *Pseudomona*s sp. CS3 was 7.0. The suitable pH ranges for nicotine catabolism of *Acinetobacte*r sp. TW was between 7.0 and 8.0, compared to a range from 6.0 to 7.0 for *Sphingomonas* sp. TY. Similarly, stable degradation of nicotine by *Pseudomonas geniculata* N1 was observed at pH 6.0–7.5 [77]. In order to improve the degradation efficiency, temperature and pH are usually optimized together. For example, the optimum temperature for the degradation of nicotine by *Ochrobactrum intermedium* DN2 is 30–37 °C, the optimum pH is 7.0, and the average degradation rate of nicotine reaches 140.5 mg/l/h under these optimal conditions [78].

In addition, in the fermentation process, the optimization of dissolved oxygen levels and medium composition is critical for enhancing the efficiency of microbial nicotine degradation. Dissolved oxygen levels directly influence microbial metabolic activity, particularly under aerobic conditions. Studies have shown that *Pseudomonas putida* S16 achieves the highest nicotine degradation rate of 160 mg/L/h at a dissolved oxygen level of 50%, while the rate significantly drops to 70 mg/L/h when the oxygen level decreases to 20% [48]. Similarly, in cultures of *Pseudomonas fluorescens*, increasing the dissolved oxygen level from 20 to 60% resulted in approximately a 50% increase in nicotine degradation rate, along with a 40% reduction in the accumulation of intermediate metabolites [30]. Simultaneously, the degradation efficiency can also be significantly improved by adjusting the carbon and nitrogen sources as well as other auxiliary components in the medium. Shu et al. [46] reported that 3 g L^−1^ nicotine could be completely degraded within 5 h when the nicotine degradation experiment was carried out in 0.05 M sodium phosphate buffer (pH 7.0). By contrast, when degradation was performed with distilled water, it took more than 8 h to be fully completed. At the same time, a number of inorganic salts were also added to the experiment to observe their effects on the nicotine degradation rate. It was found that ZnSO_4_⋅7H_2_O and NiCl_2_⋅6H_2_O had little effect on nicotine degradation, while Na_2_MoO_4_ and CuCl_2_–4H_2_O would inhibit the degradation of nicotine [79]. Yeast extract, glucose and Tween 80 are also noteworthy [79]. In addition, glucose is an important carbon source for promoting cell growth and increasing the rate of nicotine degradation [79–81]. Zhao et al. and Huang et al. found that 1 g L^−1^ glucose can enhance nicotine degradation, although glucose had no effect in solid-state fermentation experiments [79, 82]. Similarly, *Agrobacterium* sp. strain S33, isolated from tobacco rhizosphere soil (Shandong, P.R. China), which can completely degrade 1.0 g L^−1^ nicotine in 6 h at 30 °C and pH 7.0, did not completely degrade nicotine within 10 h when grown on glucose–ammonium medium [39]. This means that the fermentation conditions should be tailored to each specific strain or consortium. By comprehensively optimizing factors such as temperature, pH, dissolved oxygen levels, osmotic pressure, and medium composition, the efficiency of microbial nicotine degradation during fermentation can be significantly enhanced, thereby improving the overall quality of tobacco leaves and products. These optimization strategies not only lay a solid foundation for large-scale industrial applications but also present potential for further refinement in future research.

## Conclusions and perspectives

Nicotine is the most important alkaloid in tobacco, largely the quality of cigarettes and other tobacco products. Nicotine plays a critical role in smoking addiction and is well known to be harmful to human health, because it easily crosses the blood–brain barrier and biological membranes [83]. At the same time, the production and consumption of large amounts of tobacco products generates toxic and hazardous waste containing high concentrations of nicotine and other alkaloids, which also cause serious environmental problems due to a lack of recycling methods [32, 33]. According to EU regulations, these wastes are classified as "toxic and hazardous" when the nicotine content exceeds 0.5 g kg^−1^ [81, 84]. If left untreated, these wastes have a high potential to cause harm to human health and the environment. Nicotine has a relatively stable chemical structure, and if physical or chemical methods are used to remove nicotine, the cost will be high, there will be many harmful by-products, and sometimes even the quality of treated tobacco products will deteriorate. By contrast, microbial methods can specifically degrade nicotine [38], offering advantages in cost, efficiency, and sustainability [85].

Over the past few decades, increasing numbers of nicotine-degrading microorganisms have been isolated and identified from tobacco planting soil, tobacco leaves and tobacco waste, mainly including species of *Pseudomonas*, *Arthrobacter*, *Acinetobacter*, *Agrobacterium*, *Sinorhizobium*, and *Sphingomonas*, many of which showed high activity in degrading nicotine in tobacco leaves. Based on these early findings, researchers have conducted numerous studies on the metabolic pathways and related mechanisms of nicotine degradation by microorganisms and have successfully elucidated the pathways of several classical nicotine-degrading microorganisms, such as *A. nicotinovorans* [18, 86, 87], *P. putida* [18, 46, 58, 71], and *A. tumefaciens* [88, 89].

**Fig. 5:**
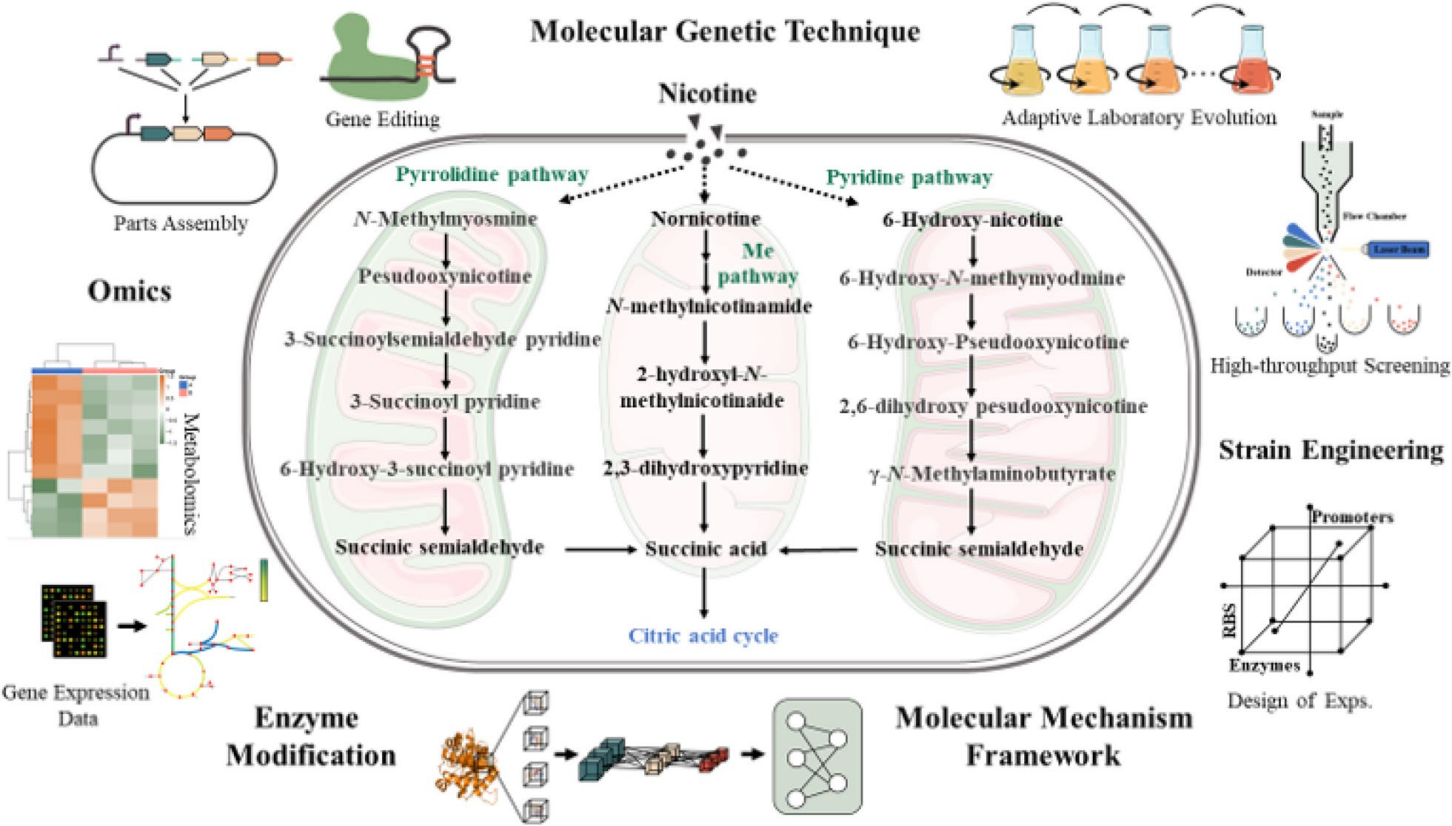
Overview of potential strategies for enhancing microbial nicotine degradation through genetic modification and metabolic engineering

Furthermore, with the growing use of nicotine-degrading microorganisms, they have been found to show potential not only in reducing the content of nicotine, but several intermediates in the nicotine degradation pathway are important precursors for the synthesis of biologically active compounds [90]. Particularly, in *Pseudomonas spp*., nicotine is metabolized to produce 6-hydroxy-3-succinyl pyridine, which can be used in the synthesis of the insecticide imidacloprid and antiparkinsonian agents, as well as 2,5-dihydroxypyridine, which can be used in the synthesis of plant growth hormones and vitamin B12 [91]. However, although microbial degradation has become a major tool in the treatment of nicotine contamination, it is often limited to a few strains, such as *Pseudomonas* sp. HF-1 [39, 92, 93], *P. putida* S16 [94], *Pseudomonas* sp. Nic22 [38] and *Acinetobacter sp.* TW [95]. As research continues, the mining of new nicotine-degrading microorganisms and the molecular biology of microbial metabolism of nicotine will be the future research trends in this field (Fig. 5). However, it should be noted that much of the research has been done on the small-scale laboratory level, and it is expected that the large-scale industrial application of nicotine degrading microorganisms for tobacco product quality improvement, tobacco waste treatment or even the conversion of nicotine to produce high value-added compounds will become a research hot spot in the future. Moreover, although some metabolic pathways have been elucidated, there are still no specific strategies for effectively improving the quality of tobacco products while reducing the harmful effects of nicotine and its metabolites on humans and the environment. Therefore, it is an urgent research challenge to combine genetic modification and metabolic engineering with abundant nicotine-degrading microbial resources to achieve these goals.

## Data Availability

The data and materials generated during and/or analyzed during the current study are publicly available.
